# Elucidating the causal association between gut microbiota and intrahepatic cholangiocarcinoma through Mendelian randomization analysis

**DOI:** 10.3389/fmicb.2023.1288525

**Published:** 2023-11-14

**Authors:** Zhitao Chen, Weiguang Shi, Kailei Chen, Chicheng Lu, Xinyuan Li, Qiyong Li

**Affiliations:** ^1^Department of Hepatobiliary Surgery, Shulan (Hangzhou) Hospital Affiliated to Zhejiang Shuren University Shulan International Medical College, Hangzhou, China; ^2^Department of Hepatobiliary Surgery, Shulan (Anji) Hospital, Anji, China; ^3^School of Medicine, Zhejiang Shuren University, Hangzhou, China; ^4^School of Medicine Zhejiang Chinese Medical University Zhejiang Shuren College, Hangzhou, China

**Keywords:** intrahepatic cholangiocarcinoma, Mendelian randomization, gut microbiota, bioinformatics analysis, tumor immune microenvironment

## Abstract

**Background:**

Intrahepatic cholangiocarcinoma (ICC) is an aggressive liver cancer with poor prognosis. The gut microbiota has been linked to ICC, but evidence for causality is lacking. Elucidating causal gut microbiota-ICC links could inform prevention and treatment strategies.

**Materials and methods:**

We performed a bidirectional two-sample Mendelian randomization (MR) study to investigate causal associations between gut microbiota and ICC risk. Genome-wide significant single nucleotide polymorphisms (SNPs) associated with gut microbiota abundances were utilized as instrumental variables (IVs). Multiple methods assessed causality and sensitivity analyses evaluated result robustness. Bioinformatics analysis of genetic loci linked to gut microbiota and ICC examined potential mechanisms.

**Results:**

Genetically predicted increases in *Veillonellaceae*, *Alistipes*, *Enterobacteriales*, and *Firmicutes* were suggestively associated with higher ICC risk, while increases in *Anaerostipes*, *Paraprevotella*, *Parasutterella*, and *Verrucomicrobia* appeared protective. Bioinformatics analysis revealed differentially expressed genes near gut microbiota-associated loci may influence ICC through regulating pathways and tumor immune microenvironment.

**Conclusion:**

Our findings provide suggestive evidence for causal links between specific gut microbiota and ICC risk.

## Introduction

Intrahepatic cholangiocarcinoma (ICC) is an aggressive cancer arising from biliary differentiation (Valle et al., 2021). Globally, it ranks as the second most prevalent form of liver cancer, accounting for roughly 15% of all primary liver malignancies (Massarweh and El-Serag, 2017). In 2022, approximately 30,000 lives are expected to be claimed by liver cancer, with ICC contributing to approximately 20% of these deaths and displaying a discouraging 5-year survival rate of less than 20% (Wang et al., 2022). ICC commonly arises in the context of persistent inflammation, which leads to cholestasis and damage to cholangiocytes. Established risk factors for ICC include hepatolithiasis, sclerosing cholangitis, viral hepatitis, obesity-related steatohepatitis (Khan et al., 2019; Labib et al., 2019). Surgery remains the only potentially curative option for ICC patients, although it is suitable for only 20–30% of individuals, and there is a high rate of tumor recurrence (Moris et al., 2023). Currently, comprehensive treatment strategies for ICC have extremely limited efficacy. This underscores an urgent need to elucidate novel mechanisms underlying ICC pathogenesis and progression, in order to develop more effective therapies against this aggressive malignancy.

Recent studies have revealed close associations between human commensal microbes and complex diseases such as mental disorders, cardiovascular diseases, and cancer (Xiao et al., 2022; Bendriss et al., 2023; Chen et al., 2023). Modulating the gut microbiota and enhancing gut barrier function has emerged as a promising new approach for preventing and treating certain diseases (Koning et al., 2023). The direct anatomical connection between the bile duct and intestinal tract via bile secretion pathways suggests potential relevance in elucidating specific associations between biliary tract disease and the gut microbiota. Cholangiocytes are continuously exposed to a wide range of commensal microbes and microbe-associated molecules that can profoundly influence the homeostasis of the cholangiocyte microenvironment. Several clinical studies have reported an increased presence of *Helicobacter species* in stool samples from patients infected with *Opisthorchis viverrini*. Specifically, they have observed the overexpression of *Helicobacter* genes, CagA and CagE. The proteins produced by these genes traverse the plasma membrane, initiating the phosphorylation of sarcoma family kinases, which may act as signaling molecules, thereby promoting fibrosis and inflammation of the bile ducts. *In vitro* experiments have demonstrated that co-culturing cholangiocarcinoma (CCA) cells with CagA-positive *Helicobacter species* leads to higher expression levels of the antiapoptotic factor Bcl-2 and the activation of mitogen-activated protein kinase and nuclear factor-kappa B (NF-κB) signaling pathways, resulting in the further proliferation of bile duct cancer cells. Additionally, numerous investigations have underscored the pivotal role of gut microbiota in upholding the integrity of the intestinal mucosal barrier and fostering the evolution and maturation of the immune system (Nagashima et al., 2023). However, the extent of research into the intricate interplay between gut microbiota and ICC is still rather limited. Consequently, a comprehensive understanding of the intricate mechanisms through which the gut microbiota exerts its influence on the genesis and therapeutic avenues of ICC warrant an in-depth exploration.

Increasing evidence highlights the interrelation between gut microbiota and ICC, however, establishing a definitive cause-and-effect relationship remains elusive. Further research is warranted to establish causal relationships and elucidate the underlying mechanisms by which the gut microbiota may influence disease, in order to provide novel insights into potential gut microbiota-targeted therapeutic strategies. The Mendelian randomization (MR) employs genetic variants derived from genome-wide association studies (GWAS) as instrumental variables (IVs) to infer the causal implications of environmental exposure on the observed outcomes. Since an individual’s genotype is established at conception and remains fixed throughout their life, there is no potential for reverse causation or confounding bias to influence the relationship between genotype and disease (Bowden and Holmes, 2019). This unique characteristic of genetic makeup ensures that any observed associations between specific genetic variants and diseases are less susceptible to the issues of causality being misinterpreted or distorted by external factors. In this current investigation, a two samples MR analysis was conducted with the aim of probing the inherent causal connections between gut microbiota and the occurrence of ICC.

## Materials and methods

### Data sources

The GWAS data for ICC was sourced from a large-scale meta-analysis conducted by Jiang et al. (2021)^1^ in a European population, which included 456,348 individuals, 11,842,647 variants and 2,989 binary traits. The analysis of the gut microbiota was performed by the Microbiome Genome (MiBioGen)^2^ Consortium, encompassing a cohort of 18, 473 individuals (24 cohorts) from various countries with 122,110 loci of variation (Kurilshikov et al., 2021). The majority of participants exhibited European ancestry, with a total of 13,266 individuals (72.3%) included in this group. A comprehensive tally of 211 taxa was systematically classified across five distinct biological categories, encompassing 9 *phyla*, 16 *classes*, 20 *orders*, 35 *families*, and 131 *genera*. Notably, 15 unidentified taxa lacking definitive taxonomic classification were excluded from the analysis, as these ambiguous groups cannot provide meaningful biological insights into potential causal relationships with disease outcomes. Ultimately, this resulted in the inclusion of 196 well-defined taxonomic units (comprising 9 *phyla*, 16 *classes*, 20 *orders*, 32 *families*, and 119 *genera*) in the present study. The details of the data sources in present MR study are shown in Table 1.

**TABLE 1:** Details of the genome-wide association studies and datasets used in our analyses.

Exposure or outcome	Sample size	Ancestry	Links for data download	PMID
Human gut microbiome	18,340 participants	Mixed (72.3% European)	https://www.ebi.ac.uk/gwas/	33462485
Intrahepatic Cholangiocarcinoma	456348 participants	European	https://www.ebi.ac.uk/gwas/	34737426

PMID, PubMed Identifier.

### Selection of IVs

Firstly, single nucleotide polymorphisms (SNPs) meeting the locus-wide significance criterion of *P* < 1 × 10^–05^ were meticulously chosen as prospective IVs associated with the gut microbiota. Secondly, to procure independent IVs from distinct loci, a linkage disequilibrium (LD) threshold of *R*^2^ < 0.001 and a clumping distance of 10,000 kb were employed in the analysis of 1000 Genomes EUR dataset. Thirdly, strict adherence was maintained to the principle of selecting SNPs with consistent allele effects on both the exposure and outcome variables. Accordingly, palindromic SNPs devoid of A/T or C/G polymorphisms were deliberately excluded from the pool of IVs. Finally, we extracted the summary data of the IVs on the health indicator under study and used the F statistics (*F* = beta^2^/se^2^) to assess the strength of the IVs (Burgess and Thompson, 2011). A value greater than 10 was considered indicative of a powerful instrument.

### Sensitivity analysis

The detection of heterogeneity between the two samples was carried out using Cochran’s *Q*-test (Bowden et al., 2018), applying both the Inverse Variance Weighted (IVW) and MR Egger (MRE) methods (Burgess and Thompson, 2017). A significance level of *p* < 0.05 was considered as indicative of the presence of heterogeneity. The application of MR-PRESSO aimed to mitigate the influence of horizontal pleiotropy through identification and elimination of potential outliers (Verbanck et al., 2018). Furthermore, a leave-one-out sensitivity analysis was conducted to affirm the robustness of the findings, systematically excluding individual SNPs with each iteration. Scatter plots and funnel plots were generated to provide a visual interpretation of the outcomes derived from the MR analyses and to discern any potential outliers within the data.

### MR analysis

A comprehensive MR analysis was conducted to ascertain the potential causal association between the gut microbiota and the susceptibility to ICC. This investigation encompassed a range of statistical methods, including the IVW, MRE, Weighted Median (WMed), Weighted Mode (WMod), and Simple Mode (SMod) methods. Consistent causal effects across multiple methods strengthen confidence in the results and conclusions. Contrasting methods also help pinpoint outliers and biases. Employing a range of methods enables assessing the robustness of the findings and ensures invalid instruments or pleiotropy do not lead to spurious conclusions. The entirety of data analyses was executed utilizing RStudio (Version: 2023.06.1 + 524) in conjunction with the Two Sample MR package (version 0.5.7). MR-PRESSO analysis was conducted employing the R package “MRPRESSO” (version 1.0).

### Reverse MR

In this study, we will perform a reverse MR analysis employing a set of gut microbiota that have been established as causally related to ICC. The objective is to mitigate the potential influence of reverse causality, thereby enhancing the trustworthiness of our research findings.

### Bioinformatics analysis

To explore the potential mechanisms underlying the role of gut microbiota in the development of ICC, we performed a bioinformatics analysis using RStudio and utilized online databases to identify genes enriched with strongly correlated genetic loci that are shared between gut microbiota and ICC. First, we utilized the NCBI database^3^ to identify putative candidate genes corresponding to the SNPs found to be shared between gut microbiota traits and ICC. We conducted a comparative analysis of miRNA expression between ICC and non-cancerous tissues utilizing the GEPIA online database,^4^ which is based on data from The Cancer Genome Atlas (TCGA) and the Genotype-Tissue Expression (GTEx) project. In order to comprehensively investigate the role of differentially expressed genes in ICC, we utilized the STRING^5^ database to analyze genes that are correlated with these differentially expressed genes for Kyoto Encyclopedia of Genes and Genomes (KEGG) pathway analysis. GSCA,^6^ an integrated platform for genomic, pharmacogenomic, and immunogenomic gene set cancer analysis, offers a comprehensive resource. By merging clinical data and information about small molecule drugs, researchers can identify potential biomarkers and promising therapeutic agents, facilitating enhanced experimental planning and subsequent clinical trials. In our study, GSCA was instrumental in revealing the correlation between drug sensitivity and immune cell interactions associated with genes enriched in strongly correlated genetic loci shared between gut microbiota and ICC.

## Results

### Identification of IVs for MR analysis

**FIGURE 1:**
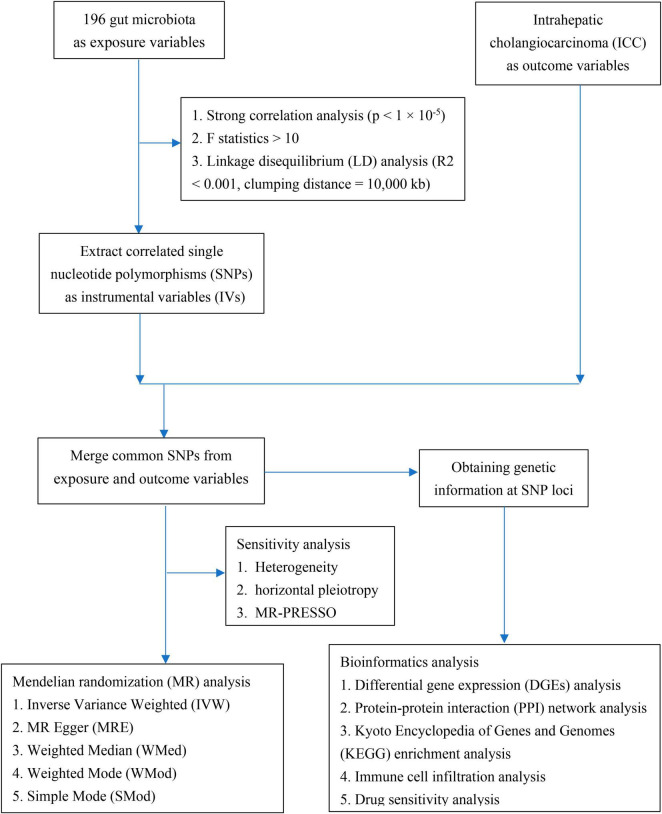
The directed acyclic graph of the present study.

The directed acyclic graph of the present study is depicted in Figure 1. Initially, a comprehensive set of 2591 SNPs, corresponding to 196 distinct taxonomic units of the gut microbiota, were extracted. These selections were made based on the stipulated threshold for locus-wide statistical significance (*P* < 1 × 10^–5^) and the LD threshold (*R*^2^ < 0.001, with a clumping distance of 10,000 kb). It was observed that all IVs exhibited an F-statistic surpassing the threshold of 10, signifying the absence of substantial indications of weak instrument bias. Comprehensive details regarding the IVs across various categories of gut microbiota are meticulously presented in Supplementary Table 1.

### Causal influence of gut microbiota on ICC

**FIGURE 2:**
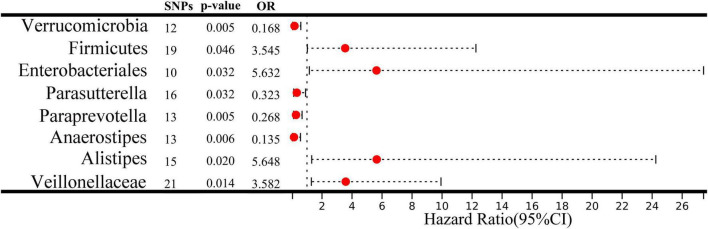
A forest plot depicts the associations between genetically predicted increases in 8 bacterial taxa and intrahepatic cholangiocarcinoma (ICC) risk. CI, confidence interval; OR, odds ratio; SNP, single nucleotide polymorphism.

**FIGURE 3:**
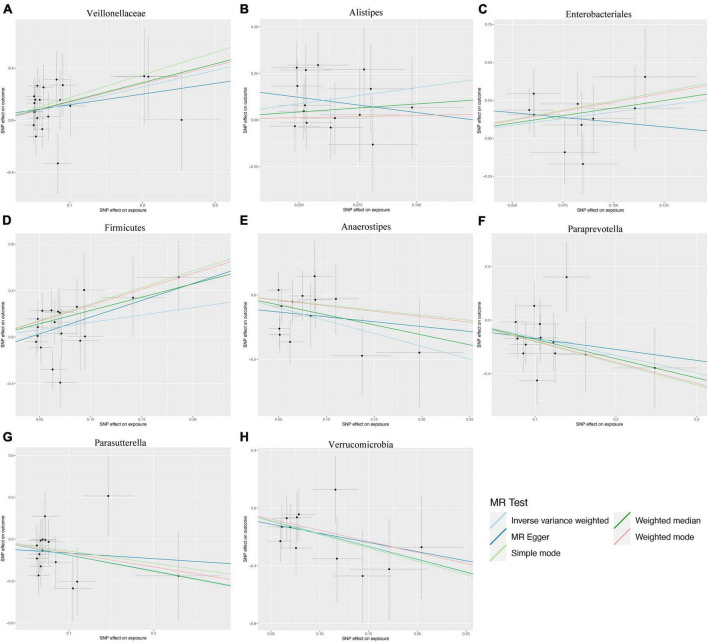
Scatter plots illustrating the genetic associations with eight bacterial taxa and intrahepatic cholangiocarcinoma (ICC). **(A)** Veillonellaceae, **(B)** Alistipes, **(C)** Enterobacteriales, **(D)** Firmicutes, **(E)** Anaerostipes, **(F)** Paraprevotella, **(G)** Parasutterella, **(H)** Verrucomicrobia.

A two-sample MR analysis was systematically executed to assess the potential causal linkage between individual categories of gut microbiota and the occurrence of ICC. Using the IVW method, we found suggestive evidence for a causal association between genetically predicted increases in *Veillonellaceae* (OR = 3.582; 95% CI: 1.292–9.929; *P* = 0.014), *Alistipes* (OR = 5.648; 95% CI: 1.316–24.245; *P* = 0.020), *Enterobacteriales/Enterobacteriaceae* (OR = 5.632; 95% CI: 1.156–27.429; *P* = 0.032), and *Firmicutes* (OR = 3.545; 95% CI: 1.025–12.258; *P* = 0.046) and higher risk of ICC, while genetically predicted increases in *Anaerostipes* (OR = 0.135; 95% CI: 0.033–0.564; *P* = 0.006), *Paraprevotella* (OR = 0.268; 95% CI: 0.107–0.672; *P* = 0.005), *Parasutterella* (OR = 0.323; 95% CI: 0.115–0.907; *P* = 0.032) and *Verrucomicrobia* (OR = 0.168; 95% CI: 0.048–0.588; *P* = 0.005) appeared to confer protective effects against ICC (Figure 2). Among the traits mentioned earlier, the *Enterobacteriales* and the *Enterobacteriaceae* were observed to belong to the same bacterial category, with identical IVs. Moreover, the MRE, WMed, SMod, and WMod yielded causal effect estimates exhibiting comparable magnitudes and directions to those obtained from the previously mentioned IVW method (Supplementary Table 2 and Figure 3). In our analysis, we have observed that while some *p*-values are below the conventional significance threshold of 0.05, the corresponding false discovery rate (FDR) values are above this threshold. Our rationale for choosing to interpret the results based on *p*-values stems from the specific context of our study. We recognize the importance of FDR correction in controlling for multiple comparisons, but believe that in this particular context, *p*-values remain a relevant and informative metric, especially when the assessment of significance requires a more conservative approach.

### Sensitivity analysis

**FIGURE 4:**
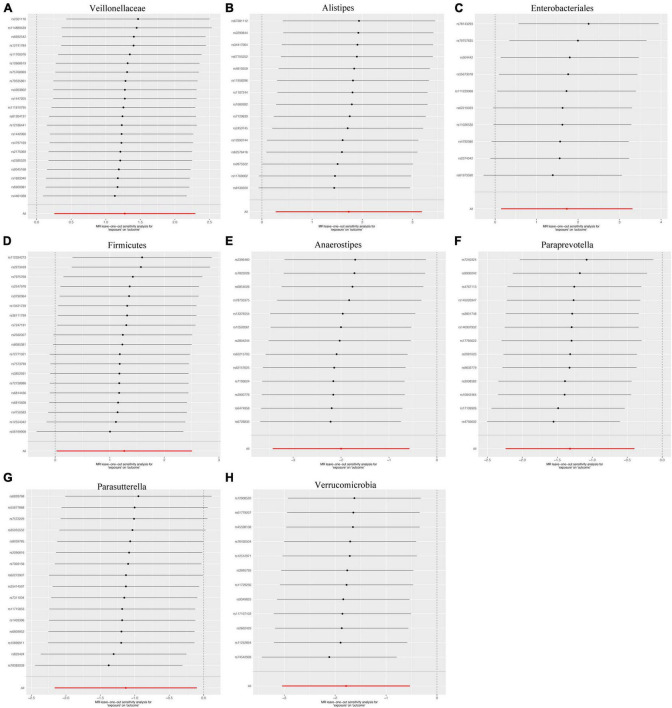
Leave-one-out the sensitivity analysis plot-the causal effect of eight bacterial taxa on intrahepatic cholangiocarcinoma (ICC). **(A)** Veillonellaceae, **(B)** Alistipes, **(C)** Enterobacteriales, **(D)** Firmicutes, **(E)** Anaerostipes, **(F)** Paraprevotella, **(G)** Parasutterella, **(H)** Verrucomicrobia.

We applied Cochran’s Q statistics utilizing IVW and MRE methodologies to assess heterogeneity. The outcomes revealed no significant heterogeneity among the IVs (all *p*-values > 0.05, Table 2). Additionally, both the MR-Egger intercept and the MR-PRESSO global test substantiated the absence of statistically significant directional horizontal pleiotropy (all *p*-values > 0.05, Table 2). Moreover, the leave-one-out analysis demonstrated the absence of influential IVs that would yield a noteworthy impact on the outcome if retained (Figure 4). These conducted sensitivity analyses, encompassing Cochran’s Q statistics, MR-Egger intercept, MR-PRESSO global test, and leave-one-out analysis, collectively showcased the robustness of the two samples MR findings. Furthermore, the funnel plot and forest plots are presented to visualize a symmetrical pattern, indicating the reliability of the results (Supplementary Figures 1, 2).

**TABLE 2:** The heterogeneity and pleiotropy analysis of the MR study on gut microbiota and ICC.

Exposure	Outcome	Method	Heterogeneity		Horizontal pleiotropy		MR-PRESSO
			**Q**	**Q_*p*-value**	**Egger intercept**	***p*-value**	***p*-value**
Verrucomicrobia	ICC	MRE	4.795	0.904	−0.048	0.762	0.951
		IVW	4.892	0.936			
Firmicutes	ICC	MRE	16.151	0.513	−0.117	0.342	0.527
		IVW	17.108	0.516			
Enterobacteriales	ICC	MRE	5.600	0.692	0.231	0.433	0.725
		IVW	6.282	0.711			
Parasutterella	ICC	MRE	9.901	0.769	−0.062	0.638	0.805
		IVW	10.133	0.811			
Paraprevotella	ICC	MRE	10.402	0.495	−0.057	0.740	0.619
		IVW	10.518	0.571			
Anaerostipes	ICC	MRE	9.312	0.593	−0.095	0.526	0.679
		IVW	9.740	0.639			
Alistipes	ICC	MRE	8.587	0.803	0.203	0.373	0.808
		IVW	9.437	0.802			
Veillonellaceae	ICC	MRE	13.399	0.818	0.037	0.672	0.850
		IVW	13.584	0.851			

MR, Mendelian randomization; ICC, intrahepatic cholangiocarcinoma; Q, Cochran’s Q-test; MRE, MR egger; IVW, inverse variance weighted.

### The result of reverse MR analysis

Finally, we assessed the possibility of reverse associations between these bacterial traits and ICC through reverse MR analyses. A total of 15 IVs were identified based on the specified threshold for locus-wide statistical significance (*P* < 1 × 10^–5^), the LD threshold (*R*^2^ < 0.001, with a clumping distance of 10,000 kb), and an F-statistic exceeding the threshold of 10 (Supplementary Table 3). Using the IVW method, we did not uncover statistically significant associations between ICC and any of these bacterial traits (Supplementary Table 4).

### Differential gene expression analysis of near genetic loci

**FIGURE 5:**
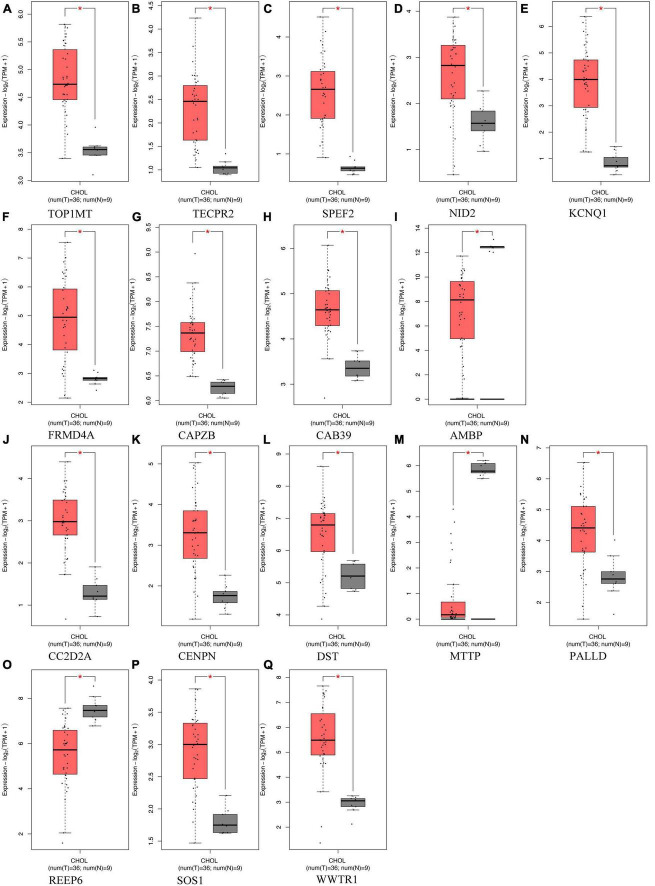
Differentially expressed genes located in proximity to genetic loci associated with both gut microbiota and intrahepatic cholangiocarcinoma (ICC) expression, comparing their expression in ICC tumor tissues (depicted in red) vs. normal tissues (depicted in gray). **(A-I)** Genes associated with gut microbiota promoting ICC formation, **(J–Q)** Genes linked to gut microbiota inhibiting ICC formation. *Indicates statistical significance at *p* < 0.05.

To acquire a more profound comprehension of the association between gut microbiota and ICC, we carried out an extensive analysis of the genetic loci linked to both gut microbiota and ICC (Supplementary Table 5). Analysis of RNA-seq data encompassing 36 ICC samples and corresponding paraneoplastic tissues sourced from TCGA and GTEx databases unveiled notable disparities in the expression of 17 genes. Among these genes, nine were associated with gut microbiota that promotes ICC, including *Veillonellaceae* (TECPR2), *Alistipes* (TOP1MT and CAPZB), *Enterobacteriales* (KCNQ1), and *Firmicutes* (AMBP, NID2, CAB39, SPEF2, and FRMD4A). Conversely, the remaining eight genes were linked to gut microbiota with inhibitory effects on ICC, including *Anaerostipes* (SOS1, PALLD, and REEP6), *Paraprevotella* (WWTR1), *Parasutterella* (CC2D2A), and *Verrucomicrobia* (CENPN, MTTP, and DST). All of these genes exhibited statistically significant differences (Figure 5). These differentially expressed genes may play a significant role in the development of ICC.

### Protein-protein interaction (PPI) network and KEGG pathway analysis

**FIGURE 6:**
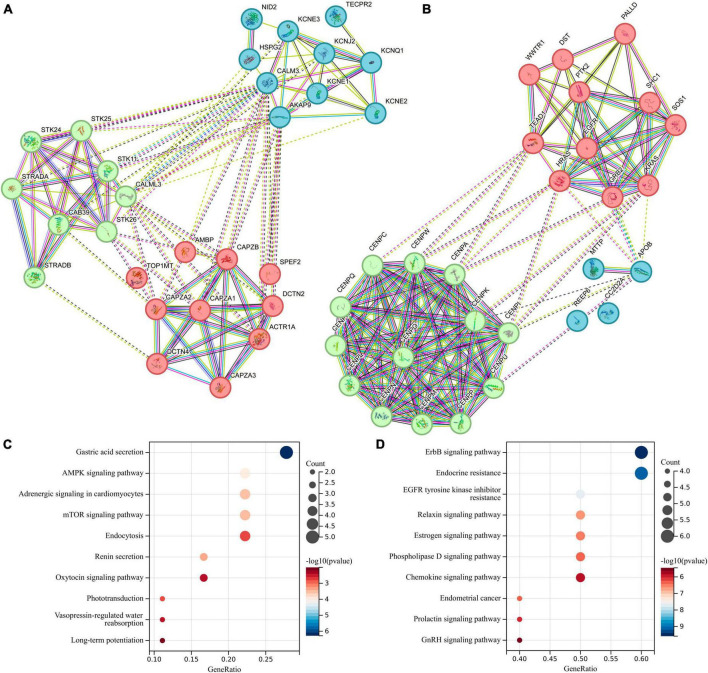
Protein-Protein Interaction (PPI) network and Kyoto Encyclopedia of Genes and Genomes (KEGG) pathway analysis for differentially expressed genes located in proximity to genetic loci associated with both gut microbiota and intrahepatic cholangiocarcinoma (ICC). **(A)** PPI network for genes associated with gut microbiota promoting ICC formation. **(B)** PPI network for genes linked to gut microbiota inhibiting ICC formation. **(C)** KEGG for genes associated with gut microbiota promoting ICC formation. **(D)** KEGG for genes linked to gut microbiota inhibiting ICC formation.

To explore the differences in functionality and pathways between these two groups of genes, we conducted a comparative analysis involving the PPI network and KEGG pathway analysis. The PPI network of the differentially expressed genes is depicted in Figures 6A, B, and we further investigated the top 20 genes with the strongest interactions among them. These genes are divided into three clusters based on their functional associations. KEGG pathway analysis was also performed to predict the altered pathways linked to two groups of genes. The KEGG pathway analysis revealed that genes associated with gut microbiota promoting ICC formation were primarily enriched in pathways related to Gastric acid secretion, AMPK signaling pathway, and mTOR signaling pathway (Figure 6C). In contrast, genes linked to gut microbiota inhibiting ICC formation were predominantly enriched in pathways such as ErbB signaling pathway, Endocrine resistance, and EGFR tyrosine kinase inhibitor resistance, as indicated by our KEGG pathway analysis (Figure 6D).

### Immune cell infiltration analysis and drug sensitivity analysis

**FIGURE 7:**
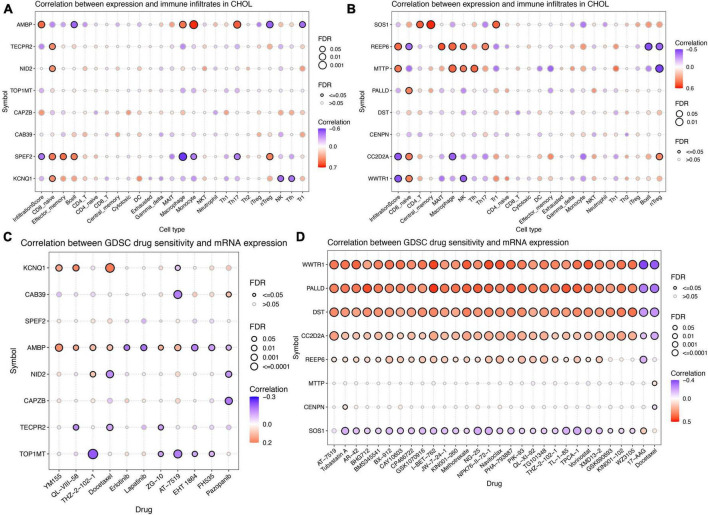
Immune cell infiltration analysis and drug sensitivity analysis for differentially expressed genes located in proximity to genetic loci associated with both gut microbiota and intrahepatic cholangiocarcinoma (ICC). **(A)** Immune cell infiltration analysis for genes associated with gut microbiota promoting ICC formation. **(B)** Immune cell infiltration analysis for genes linked to gut microbiota inhibiting ICC formation. **(C)** Drug sensitivity analysis for genes associated with gut microbiota promoting ICC formation. **(D)** Drug sensitivity analysis for genes linked to gut microbiota inhibiting ICC formation.

To explore the role of differentially expressed genes in shaping the tumor immune microenvironment during tumor progression, we conducted an analysis of immune cell infiltration in ICC as outlined in scholarly literature. In the genome of gut microbiota that promotes ICC development, AMBP exhibits a significant positive correlation with Infiltration Score, significantly promoting the infiltration of Monocytes and Th17 cells in ICC tissues (Figure 7A). Conversely, SPEF2 displays a significant negative correlation with Infiltration Score and significantly inhibits the infiltration of Macrophages in ICC tissues (Figure 7A). In the genomic context of gut microbiota that inhibits ICC development, both REEP6 and MTTP exhibit a significant positive correlation with Infiltration Score, significantly promoting the infiltration of Mucosal-Associated Invariant T cells (MAIT), Macrophages, as well as Natural Killer (NK) cells, and Follicular Helper T (Tfh) cells in ICC tissues (Figure 7B). Conversely, CC2D2A and WWTR1 display a significant negative correlation with Infiltration Score and significantly inhibit the infiltration of Macrophages and NK cells in ICC tissues (Figure 7B). In order to investigate the drug sensitivity of genetic loci associated with both gut microbiota and ICC, we performed a drug sensitivity analysis utilizing the GDSC database (Figures 7C, D). In genes associated with gut microbiota that promote ICC, we found a positive correlation between KCNQ1 expression and sensitivity to YM155, QL-VIII-58, and Docetaxel; CAB39 showed a negative correlation with AT-7519; AMBP displayed a positive correlation with YM155, QL-VIII-58, THZ-2-102-1, Docetaxel, ZG-10, and AT-7519, while it exhibited a negative correlation with Erlotinib, Lapatinib, EHT 1864, FH535, and Pazopanib; NID2 had a significant negative correlation with Docetaxel and Pazopanib; TECPR2 showed a negative correlation with QL-VIII-58, Docetaxel, and ZG-10. TOP1MT displayed a negative correlation with THZ-2-102-1, ZG-10, AT-7519, EHT 1864, and FH535. In genes associated with gut microbiota that inhibit ICC, the expression of WWTR1, REEP6, CC2D2A, DST, and PALLD is positively correlated with sensitivity to AT-7519, Tubastatin A, AR-42, BHG712, BMS345541, BX-912, CAY10603, CP466722, GSK1070916, I-BET-762, JW-7-24-1, KIN001-260, Methotrexate, NG-25, NPK76-II-72-1, Navitoclax, PHA-793887, PIK-93, QL-XI-92, TG101348, THZ-2-102-1, TL-1-85, TPCA-1, Vorinostat, and XMD13-2, while it is negatively correlated with 17-AAG and Docetaxel. However, the results for SOS1 are the opposite.

## Discussion

Intrahepatic cholangiocarcinoma is an insidious form of liver cancer that has been exhibiting a rising incidence globally (Clements et al., 2020). Despite accounting for only 15% of primary liver malignancies, ICC arising from the biliary epithelium represent a major and growing threat to public health worldwide (Beal et al., 2018; Banales et al., 2019; Khan et al., 2019). The human intestine harbors a complex gut microbiota comprising bacteria, fungi, archaea, viruses, and protozoa that plays a vital role in maintaining human health (Jandhyala et al., 2015). This gut microbiota exists in symbiosis with the gut mucosa and provides critical immunologic, metabolic, and gastrointestinal protective functions in healthy individuals (Wang Q. et al., 2023). A reduction in microbial biodiversity within the gut microbiota could elevate susceptibility to diverse diseases, including the development of malignancies such as cancers (Lee et al., 2023; Rajapakse et al., 2023). Similarly, there has been substantial research concerning the role of the gut microbiota in the occurrence and progression of ICC, as well as its implications for diagnosis and treatment strategies (Zhang et al., 2022; Pomyen et al., 2023). Nonetheless, a comprehensive causal relationship analysis concerning the interplay between gut microbiota and ICC remains lacking in the current literature.

Consequently, our study first conducted a two-sample MR analysis, utilizing summary statistics from GWAS, to investigate the potential causal link between gut microbiota and ICC. This analytical approach not only holds promise for effective ICC prevention and intervention strategies but also provides innovative insights into ICC pathogenesis through the perspective of gut microbiota.

Anatomically and physiologically, the hepatobiliary duct and the gastrointestinal tract are intricately interconnected, forming a ‘gut-liver axis’ that plays a pivotal role in regulating liver pathology and influencing both intrahepatic and systemic immune responses (Gopalakrishnan et al., 2018). Consequently, the gut microbiota assumes a significant role in modulating anti-tumor immune mechanisms. Impaired intestinal barrier function, disturbances in the intestinal environment, and reduced microbial diversity along the mucosal lining have been reported in various hepatobiliary duct disorders (Li et al., 2023; Rajapakse et al., 2023). Previous research has indicated a substantial increase in *Candida albicans* abundance in ICC cases, with alterations in its composition becoming more prominent as the TNM stage of ICC advances (Zhang et al., 2022). *Candida albicans* has been shown to expedite the progression of gastrointestinal cancer through the upregulation of matrix metalloproteinases synthesis, oncometabolite production, activation of pro-tumor signaling pathways, as well as the enhancement of prognostic marker genes associated with metastatic occurrences (Talapko et al., 2023; Wang X. et al., 2023). On the other hand, *Saccharomyces cerevisiae* has been identified as a microbial population that exerts a protective role against liver injury (Lai et al., 2009; Sivignon et al., 2015). It has demonstrated the potential to impede the progression of colorectal tumor growth by facilitating epithelial cell apoptosis, modulating intestinal immunity, and altering gut microbial composition (Li et al., 2020). However, it is noteworthy that *Saccharomyces cerevisiae* is notably diminished in patients with ICC (Zhang et al., 2022).

**FIGURE 8:**
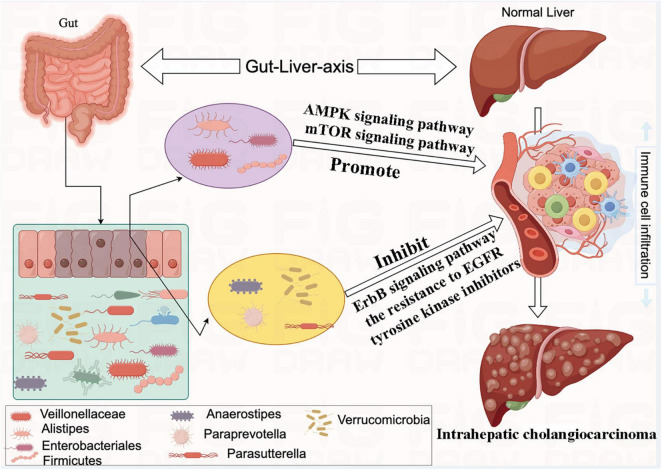
The mechanism of eight bacterial taxa and influencing the formation of intrahepatic cholangiocarcinoma (ICC).

In the present two-sample MR study, we detected suggestive causal associations between eight specific bacterial genera and the risk of ICC. Our findings provide suggestive evidence for causal associations between genetically predicted increases in the abundances of *Veillonellaceae*, *Alistipes*, *Enterobacteriales*, and *Firmicutes* and an elevated risk of ICC. In contrast, genetically predicted increases in the levels of *Anaerostipes*, *Paraprevotella*, *Parasutterella*, and *Verrucomicrobia* appeared to confer protective effects against ICC. We further constructed a schematic diagram illustrating the potential mechanisms by which these gut microbiota influences the formation of ICC (Figure 8).

Elevated abundances of *Veillonellaceae* have been identified in the intestinal microbiota of liver cancer patients (Ponziani et al., 2019; Ren et al., 2019). Increased Veillonellaceae may accelerate hepatic steatosis by producing carbohydrates and short-chain fatty acids. However, it can also generate carbon monoxide and hydrogen sulfide, which have toxic effects on both normal hepatocytes and bile duct cells, potentially contributing to the development of liver cancer (Fukui, 2019; Demir et al., 2020; Lee et al., 2023). Although *Alistipes* is predominantly found in the intestinal tract of healthy humans, it has also been isolated from the bloodstream, appendix, and abdominal regions, highlighting its potential opportunistic pathogenic role in human diseases (Shkoporov et al., 2015; Parker et al., 2020). The study also discovered that *Alistipes* could promote the development of colorectal cancer through activation of the interleukin-6/signal transducer and activator of transcription 3 signaling pathway (Feng et al., 2015). The levels of *Enterobacteriales* have been notably elevated in the intestines of patients with bacterial liver abscess, indicating a close association with its occurrence (Chen et al., 2018). A significant enrichment of *Enterobacteriales* was discovered to be associated with extended survival in cervical cancer patients undergoing chemoradiation (Sims et al., 2021). This enrichment could potentially augment the tumor infiltration of CD4 + lymphocytes, alongside activated subsets of CD4 cells expressing KI67 + and CD69 + markers throughout the course of radiation therapy (Sims et al., 2021). Yan et al. (2023) revealed dysbiosis of Firmicutes in patients with Hepatitis B Virus-Related Chronic Liver Disease (HBV-CLD), which was linked to negative regulation of liver function and T cell immune responses.

Recent studies have suggested that the gut bacterium *Anaerostipes* may confer protective effects against specific types of cancer. Notably, in murine models, a negative association between the abundance of *Anaerostipes* and the incidence of colorectal cancer is notably observed, and these protective mechanisms are believed to be linked to the production of butyrate and the enhancement of intestinal barrier function (Singh et al., 2022). This aligns with our research findings, as we have also discovered that *Anaerostipes* exerts an inhibitory role in the formation of ICC. An elevated prevalence of *Paraprevotella*, negatively correlated with hepatocellular carcinoma, may be attributed to potential mechanisms such as its anti-inflammatory properties and the inhibition of pro-carcinogenic microorganisms (Chen et al., 2015; Routy et al., 2018). Additionally, Pi et al. (2020) identified a notable presence of *Parasutterella* in colorectal cancer patients undergoing PD-1 treatment. Consequently, they propose that the PD-1/PD-L1 signaling pathway may modulate the metabolic activity of intestinal flora, including *Parasutterella*, thus enhancing immune surveillance against tumors (Pi et al., 2020). In a prior study conducted by Su et al. (2023) it was similarly observed that *Verrucomicrobia* maintained a robust negative correlation with ICC.

Our research has further revealed that *Veillonellaceae*, *Alistipes*, *Enterobacteriales*, and *Firmicutes* can promote the formation of ICC through the regulation of the AMPK signaling pathway and the mTOR signaling pathway. Meng et al. (2023) have similarly reported that alterations in the AMPK- mTOR signaling pathway can exacerbate the progression of disrupted energy metabolism, chronic inflammation, hypoxia, and cellular aging within the tumor microenvironment. These factors collectively promote the transformation of fatty liver into liver cancer. Our investigation has also unveiled that *Anaerostipes*, *Paraprevotella*, *Parasutterella*, and *Verrucomicrobia* inhibit the development of ICC through the regulation of the ErbB signaling pathway and the resistance to EGFR tyrosine kinase inhibitors. EGFR and ErbB belong to a family of cell membrane protein receptors capable of receiving external stimuli and initiating downstream signaling cascades, thus instigating a range of regulatory processes relevant to both physiological functions and pathological conditions. Studies have demonstrated that inhibiting EGFR can effectively impede hepatocellular carcinoma cell survival, migration, and invasion (Jin et al., 2021).

The role of the immune system in immunosurveillance and its control over tumor growth is now firmly established. Tumor growth and progression are frequently linked to an impaired or fatigued anti-tumor immune response (Dumauthioz et al., 2018). Previous evidence suggests distinct gut microbiota can enhance systemic and antitumor immune response (Gopalakrishnan et al., 2018). In present study, we also investigated the influence of genetic loci on the gut microbiota regarding tumor immune cell infiltration. However, the underlying mechanisms of this phenomenon require further exploration.

In terms of ICC treatment, we also explored further. We identified a gene, SOS1 (rs6726833), located near a genetic locus associated with the gut bacterium *Anaerostipes*, which showed a negative correlation with ICC. The expression of SOS1 was positively correlated with sensitivity to 17-AAG. The HSP90 inhibitor 17-AAG effectively suppressed cell growth, leading to G2/M cell cycle arrest and the induction of apoptosis in cholangiocarcinoma cells. Zhang et.al demonstrated that the inhibition of HSP90 function by 17-AAG may offer a promising therapeutic approach for treating human cholangiocarcinoma (Zhang et al., 2013). We also identified WWTR1 (rs140997932), REEP6 (rs78735375), CC2D2A (rs35414597), DST (rs9349825), and PALLD (rs6854026), which are located near genetic loci associated with *Paraprevotella*, *Anaerostipes*, *Parasutterella*, *Verrucomicrobia*, and *Anaerostipes*. CAY10603 is a highly selective acetylcholinesterase inhibitor (AChE), and its sensitivity shows a significant positive correlation with the expression levels of these genes. CAY10603, through its selective inhibition of AChE and subsequent elevation of acetylcholine levels, activates surface receptors on cholangiocarcinoma cells, inducing apoptosis in tumor cells (Khorsandi et al., 2021).

While this study provides novel suggestive evidence for causal links between specific gut microbiota and ICC risk, an important limitation is that the GWAS data utilized was primarily from populations of European ancestry. Both the GWAS data on ICC risk and the gut microbiota GWAS data had samples that were over 70% European. Thus, the results may not be fully generalizable to non-European populations. Further research in more diverse populations is needed to determine if similar microbiota-ICC associations are present across different ethnicities. Additionally, environmental and lifestyle factors that influence the gut microbiota likely vary across populations, so replication in non-European cohorts is important. In summary, although this MR analysis provides initial evidence for potential microbiota-based prevention and treatment opportunities for ICC, confirmation in multi-ethnic studies is needed before translating findings to clinical practice globally.

## Conclusion

The present two-sample MR study provides suggestive evidence for causal associations between specific gut microbiota and risk of ICC. Genetically predicted increases in *Veillonellaceae*, *Alistipes*, *Enterobacteriales*, and *Firmicutes* were associated with higher ICC risk, while increases in *Anaerostipes*, *Paraprevotella*, *Parasutterella*, and *Verrucomicrobia* appeared protective against ICC. Bioinformatics analysis revealed gut microbiota may influence ICC development through regulating pathways like AMPK, mTOR, EGFR and tumor immune microenvironment. Further research is warranted to confirm the causality and elucidate mechanisms underlying the gut microbiota-ICC link, to inform potential microbiome-targeted prevention and therapeutic strategies.

## Data availability statement

The original contributions presented in the study are included in the article/Supplementary material, further inquiries can be directed to the corresponding author.

## Author contributions

ZC: Data curation, Investigation, Methodology, Software, Writing – original draft. WS: Investigation, Methodology, Software, Writing – original draft, Writing – review and editing. KC: Data curation, Software, Writing – original draft. CL: Data curation, Formal analysis, Software, Writing – original draft. XL: Methodology, Software, Supervision, Writing – review and editing. QL: Formal analysis, Project administration, Writing – review and editing.

## Footnotes

1
https://www.ebi.ac.uk/gwas/efotraits/EFO_1001961

2
https://mibiogen.gcc.rug.nl

3
https://www.ncbi.nlm.nih.gov/

4
http://gepia.cancer-pku.cn/

5
https://string-db.org/

6
http://bioinfo.life.hust.edu.cn/GSCA/#/

## Supplementary material

Supplementary Figure 1 Forest plot of the causal effects of gut microbiota associated single nucleotide polymorphisms (SNPs) on intrahepatic cholangiocarcinoma (ICC). **(A)** Veillonellaceae, **(B)** Alistipes, **(C)** Enterobacteriales, **(D)** Firmicutes, **(E)** Anaerostipes, **(F)** Paraprevotella, **(G)** Parasutterella, **(H)** Verrucomicrobia.

Supplementary Figure 2 Funnel plot showing the relationship between the cause-effect of gut microbiota and intrahepatic cholangiocarcinoma (ICC). **(A)** Veillonellaceae, **(B)** Alistipes, **(C)** Enterobacteriales, **(D)** Firmicutes, **(E)** Anaerostipes, **(F)** Paraprevotella, **(G)** Parasutterella, **(H)** Verrucomicrobia.

## References

[B1] Banales J. Iñarrairaegui M. Arbelaiz A. Milkiewicz P. Muntané J. Muñoz-Bellvis L. (2019). Serum metabolites as diagnostic biomarkers for Cholangiocarcinoma, hepatocellular carcinoma, and primary sclerosing cholangitis. *Hepatology* 70 547–562. 10.1002/hep.30319 30325540 PMC6767196

[B2] Beal E. Tumin D. Moris D. Zhang X. Chakedis J. Dilhoff M. (2018). Cohort contributions to trends in the incidence and mortality of intrahepatic cholangiocarcinoma. *Hepatobiliary Surg. Nutr.* 7 270–276. 10.21037/hbsn.2018.03.16 30221154 PMC6131266

[B3] Bendriss G. MacDonald R. McVeigh C. (2023). Microbial reprogramming in obsessive-compulsive disorders: A review of gut-brain communication and emerging evidence. *Int. J. Mol. Sci.* 24:11978. 10.3390/ijms241511978 37569349 PMC10419219

[B4] Bowden J. Holmes M. (2019). Meta-analysis and Mendelian randomization: A review. *Res. Synth. Methods* 10 486–496. 10.1002/jrsm.1346 30861319 PMC6973275

[B5] Bowden J. Spiller W. Del Greco M. Sheehan N. Thompson J. Minelli C. (2018). Improving the visualization, interpretation and analysis of two-sample summary data Mendelian randomization via the radial plot and radial regression. *Int. J. Epidemiol.* 47 1264–1278. 10.1093/ije/dyy101 29961852 PMC6124632

[B6] Burgess S. Thompson S. (2011). Avoiding bias from weak instruments in Mendelian randomization studies. *Int. J. Epidemiol.* 40 755–764. 10.1093/ije/dyr036 21414999

[B7] Burgess S. Thompson S. (2017). Interpreting findings from Mendelian randomization using the MR-Egger method. *Eur. J. Epidemiol.* 32 377–389. 10.1007/s10654-017-0255-x 28527048 PMC5506233

[B8] Chen N. Ling Z. Jin T. Li M. Zhao S. Zheng L. (2018). Altered profiles of gut microbiota in *Klebsiella pneumoniae*-induced pyogenic liver abscess. *Curr. Microbiol.* 75 952–959. 10.1007/s00284-018-1471-7 29637226

[B9] Chen Y. Guo J. Qian G. Fang D. Shi D. Guo L. (2015). Gut dysbiosis in acute-on-chronic liver failure and its predictive value for mortality. *J. Gastroenterol. Hepatol.* 30 1429–1437. 10.1111/jgh.12932 25711972

[B10] Chen Z. Ding C. Gu Y. He Y. Chen B. Zheng S. (2023). Association between gut microbiota and hepatocellular carcinoma from 2011 to 2022: Bibliometric analysis and global trends. *Front. Oncol.* 13:1120515. 10.3389/fonc.2023.1120515 37064156 PMC10098157

[B11] Clements O. Eliahoo J. Kim J. Taylor-Robinson S. Khan S. (2020). Risk factors for intrahepatic and extrahepatic cholangiocarcinoma: A systematic review and meta-analysis. *J. Hepatol.* 72 95–103. 10.1016/j.jhep.2019.09.007 31536748

[B12] Demir M. Lang S. Martin A. Farowski F. Wisplinghoff H. Vehreschild M. (2020). Phenotyping non-alcoholic fatty liver disease by the gut microbiota: Ready for prime time? *J. Gastroenterol. Hepatol.* 35 1969–1977. 10.1111/jgh.15071 32267559

[B13] Dumauthioz N. Labiano S. Romero P. (2018). Tumor resident memory T cells: New players in immune surveillance and therapy. *Front. Immunol.* 9:2076. 10.3389/fimmu.2018.02076 30258445 PMC6143788

[B14] Feng Q. Liang S. Jia H. Stadlmayr A. Tang L. Lan Z. (2015). Gut microbiome development along the colorectal adenoma-carcinoma sequence. *Nat. Commun.* 6:6528. 10.1038/ncomms7528 25758642

[B15] Fukui H. (2019). Role of gut Dysbiosis in liver diseases: What have we learned so far? *Diseases* 7:58. 10.3390/diseases7040058 31726747 PMC6956030

[B16] Gopalakrishnan V. Spencer C. Nezi L. Reuben A. Andrews M. Karpinets T. (2018). Gut microbiome modulates response to anti-PD-1 immunotherapy in melanoma patients. *Science* 359 97–103. 10.1126/science.aan4236 29097493 PMC5827966

[B17] Jandhyala S. Talukdar R. Subramanyam C. Vuyyuru H. Sasikala M. Nageshwar Reddy D. (2015). Role of the normal gut microbiota. *World J. Gastroenterol.* 21 8787–8803. 10.3748/wjg.v21.i29.8787 26269668 PMC4528021

[B18] Jiang L. Zheng Z. Fang H. Yang J. (2021). A generalized linear mixed model association tool for biobank-scale data. *Nat. Genet.* 53 1616–1621. 10.1038/s41588-021-00954-4 34737426

[B19] Jin Q. Cheng M. Xia X. Han Y. Zhang J. Cao P. (2021). Down-regulation of MYH10 driven by chromosome 17p13.1 deletion promotes hepatocellular carcinoma metastasis through activation of the EGFR pathway. *J. Cell Mol. Med.* 25 11142–11156. 10.1111/jcmm.17036 34738311 PMC8650048

[B20] Khan S. Tavolari S. Brandi G. (2019). Cholangiocarcinoma: Epidemiology and risk factors. *Liver Int.* 39(Suppl. 1), 19–31. 10.1111/liv.14095 30851228

[B21] Khorsandi S. Dokal A. Rajeeve V. Britton D. Illingworth M. Heaton N. (2021). Computational analysis of Cholangiocarcinoma Phosphoproteomes identifies patient-specific drug targets. *Cancer Res.* 81 5765–5776. 10.1158/0008-5472.Can-21-0955 34551960 PMC9397618

[B22] Koning M. Herrema H. Nieuwdorp M. Meijnikman A. (2023). Targeting nonalcoholic fatty liver disease via gut microbiome-centered therapies. *Gut Microbes* 15:2226922. 10.1080/19490976.2023.2226922 37610978 PMC10305510

[B23] Kurilshikov A. Medina-Gomez C. Bacigalupe R. Radjabzadeh D. Wang J. Demirkan A. (2021). Large-scale association analyses identify host factors influencing human gut microbiome composition. *Nat. Genet.* 53 156–165. 10.1038/s41588-020-00763-1 33462485 PMC8515199

[B24] Labib P. Goodchild G. Pereira S. (2019). Molecular pathogenesis of Cholangiocarcinoma. *BMC Cancer* 19:185. 10.1186/s12885-019-5391-0 30819129 PMC6394015

[B25] Lai J. Hsieh W. Fang H. Lin W. (2009). The protective effects of a fermented substance from Saccharomyces cerevisiae on carbon tetrachloride-induced liver damage in rats. *Clin. Nutr.* 28 338–345. 10.1016/j.clnu.2009.01.011 19233522

[B26] Lee S. Park H. Kim C. Kim H. (2023). Dysbiosis of gut microbiota during fecal stream diversion in patients with colorectal cancer. *Gut Pathog.* 15:40. 10.1186/s13099-023-00566-9 37596621 PMC10439566

[B27] Li J. Li J. Xie Y. Wang Y. Shen X. Qian Y. (2020). Saccharomyces cerevisiae may serve as a probiotic in colorectal cancer by promoting cancer cell apoptosis. *J. Dig. Dis.* 21 571–582. 10.1111/1751-2980.12930 33245627

[B28] Li Z. Yuan H. Chu H. Yang L. (2023). The crosstalk between gut microbiota and bile acids promotes the development of non-alcoholic fatty liver disease. *Microorganisms* 11:2059. 10.3390/microorganisms11082059 37630619 PMC10459427

[B29] Massarweh N. El-Serag H. (2017). Epidemiology of hepatocellular carcinoma and intrahepatic Cholangiocarcinoma. *Cancer Control* 24:1073274817729245. 10.1177/1073274817729245 28975830 PMC5937247

[B30] Meng S. Gu H. Zhang T. Li Y. Tang H. (2023). Gradual deterioration of fatty liver disease to liver cancer via inhibition of AMPK signaling pathways involved in energy-dependent disorders, cellular aging, and chronic inflammation. *Front. Oncol.* 13:1099624. 10.3389/fonc.2023.1099624 36937390 PMC10018212

[B31] Moris D. Palta M. Kim C. Allen P. Morse M. Lidsky M. (2023). Advances in the treatment of intrahepatic cholangiocarcinoma: An overview of the current and future therapeutic landscape for clinicians. *CA Cancer J. Clin.* 73 198–222. 10.3322/caac.21759 36260350

[B32] Nagashima K. Zhao A. Atabakhsh K. Bae M. Blum J. Weakley A. (2023). Mapping the T cell repertoire to a complex gut bacterial community. *Nature* 621 162–170. 10.1038/s41586-023-06431-8 37587342 PMC10948025

[B33] Parker B. Wearsch P. Veloo A. Rodriguez-Palacios A. (2020). The genus alistipes: Gut bacteria with emerging implications to inflammation, cancer, and mental health. *Front. Immunol.* 11:906. 10.3389/fimmu.2020.00906 32582143 PMC7296073

[B34] Pi H. Huang L. Liu H. Liang S. Mei J. (2020). Effects of PD-1/PD-L1 signaling pathway on intestinal flora in patients with colorectal cancer. *Cancer Biomark.* 28 529–535. 10.3233/cbm-201606 32568184 PMC12662382

[B35] Pomyen Y. Chaisaingmongkol J. Rabibhadana S. Pupacdi B. Sripan D. Chornkrathok C. (2023). Gut dysbiosis in Thai intrahepatic cholangiocarcinoma and hepatocellular carcinoma. *Sci. Rep.* 13:11406. 10.1038/s41598-023-38307-2 37452065 PMC10349051

[B36] Ponziani F. Bhoori S. Castelli C. Putignani L. Rivoltini L. Del Chierico F. (2019). Hepatocellular carcinoma is associated with gut Microbiota profile and inflammation in nonalcoholic fatty liver disease. *Hepatology* 69 107–120. 10.1002/hep.30036 29665135

[B37] Rajapakse J. Khatiwada S. Akon A. Yu K. Shen S. Zekry A. (2023). Unveiling the complex relationship between gut microbiota and liver cancer: Opportunities for novel therapeutic interventions. *Gut Microbes* 15 2240031. 10.1080/19490976.2023.2240031 37615334 PMC10454000

[B38] Ren Z. Li A. Jiang J. Zhou L. Yu Z. Lu H. (2019). Gut microbiome analysis as a tool towards targeted non-invasive biomarkers for early hepatocellular carcinoma. *Gut* 68 1014–1023. 10.1136/gutjnl-2017-315084 30045880 PMC6580753

[B39] Routy B. Le Chatelier E. Derosa L. Duong C. Alou M. Daillère R. (2018). Gut microbiome influences efficacy of PD-1-based immunotherapy against epithelial tumors. *Science* 359 91–97. 10.1126/science.aan3706 29097494

[B40] Shkoporov A. Chaplin A. Khokhlova E. Shcherbakova V. Motuzova O. Bozhenko V. (2015). Alistipes inops sp. nov. and *Coprobacter secundus* sp. nov., isolated from human faeces. *Int. J. Syst. Evol. Microbiol.* 65 4580–4588. 10.1099/ijsem.0.000617 26377180

[B41] Sims T. El Alam M. Karpinets T. Dorta-Estremera S. Hegde V. Nookala S. (2021). Gut microbiome diversity is an independent predictor of survival in cervical cancer patients receiving chemoradiation. *Commun. Biol.* 4:237. 10.1038/s42003-021-01741-x 33619320 PMC7900251

[B42] Singh V. Lee G. Son H. Koh H. Kim E. Unno T. (2022). Butyrate producers, “The Sentinel of Gut”: Their intestinal significance with and beyond butyrate, and prospective use as microbial therapeutics. *Front. Microbiol.* 13:1103836. 10.3389/fmicb.2022.1103836 36713166 PMC9877435

[B43] Sivignon A. de Vallée A. Barnich N. Denizot J. Darcha C. Pignède G. (2015). Saccharomyces cerevisiae CNCM I-3856 prevents colitis induced by AIEC bacteria in the transgenic mouse model mimicking Crohn’s disease. *Inflamm Bowel. Dis.* 21 276–286. 10.1097/mib.0000000000000280 25569734

[B44] Su Q. Jin C. Bo Z. Yang Y. Wang J. Wang J. (2023). Association between gut microbiota and gastrointestinal cancer: A two-sample bi-directional Mendelian randomization study. *Front. Microbiol.* 14:1181328. 10.3389/fmicb.2023.1181328 37533836 PMC10390774

[B45] Talapko J. Meštrović T. Dmitrović B. Juzbašić M. Matijević T. Bekić S. (2023). A putative role of *Candida albicans* in promoting cancer development: A current state of evidence and proposed mechanisms. *Microorganisms* 11:1476. 10.3390/microorganisms11061476 37374978 PMC10305569

[B46] Valle J. Kelley R. Nervi B. Oh D. Zhu A. (2021). Biliary tract cancer. *Lancet* 397 428–444. 10.1016/s0140-6736(21)00153-7 33516341

[B47] Verbanck M. Chen C. Neale B. Do R. (2018). Detection of widespread horizontal pleiotropy in causal relationships inferred from Mendelian randomization between complex traits and diseases. *Nat. Genet.* 50 693–698. 10.1038/s41588-018-0099-7 29686387 PMC6083837

[B48] Wang Q. Lu Q. Jia S. Zhao M. (2023). Gut immune microenvironment and autoimmunity. *Int. Immunopharmacol.* 124(Pt A):110842. 10.1016/j.intimp.2023.110842 37643491

[B49] Wang X. Zhang W. Wu W. Wu S. Young A. Yan Z. (2023). Is *Candida albicans* a contributor to cancer? A critical review based on the current evidence. *Microbiol. Res.* 272:127370. 10.1016/j.micres.2023.127370 37028206

[B50] Wang Y. Strazzabosco M. Madoff D. (2022). Locoregional therapy in the management of intrahepatic Cholangiocarcinoma: Is there sufficient evidence to guide current clinical practice? *Curr. Oncol. Rep.* 24 1741–1750. 10.1007/s11912-022-01338-5 36255606 PMC10878124

[B51] Xiao M. Wan Z. Lin X. Wang D. Chen Z. Gu Y. (2022). ABO-incompatible liver transplantation under the desensitization protocol with rituximab: Effect on biliary Microbiota and metabolites. *J. Clin. Med.* 12 141. 10.3390/jcm12010141 36614942 PMC9821037

[B52] Yan F. Zhang Q. Shi K. Zhang Y. Zhu B. Bi Y. (2023). Gut microbiota dysbiosis with hepatitis B virus liver disease and association with immune response. *Front. Cell Infect. Microbiol.* 13:1152987. 10.3389/fcimb.2023.1152987 37201112 PMC10185817

[B53] Zhang J. Zheng Z. Zhao Y. Zhang T. Gu X. Yang W. (2013). The heat shock protein 90 inhibitor 17-AAG suppresses growth and induces apoptosis in human cholangiocarcinoma cells. *Clin. Exp. Med.* 13 323–328. 10.1007/s10238-012-0208-3 22955701

[B54] Zhang L. Chen C. Chai D. Kuang T. Deng W. Wang W. (2022). Alterations of gut mycobiota profiles in intrahepatic cholangiocarcinoma. *Front. Microbiol.* 13:1090392. 10.3389/fmicb.2022.1090392 36687597 PMC9853418

