# Supplementary figures and images for: Elucidating the causal association between gut microbiota and intrahepatic cholangiocarcinoma through Mendelian randomization analysis
Source: Front Microbiol. 2023 Nov 14;14:1288525. doi: 10.3389/fmicb.2023.1288525 (PMC10682188; doi:10.3389/fmicb.2023.1288525)

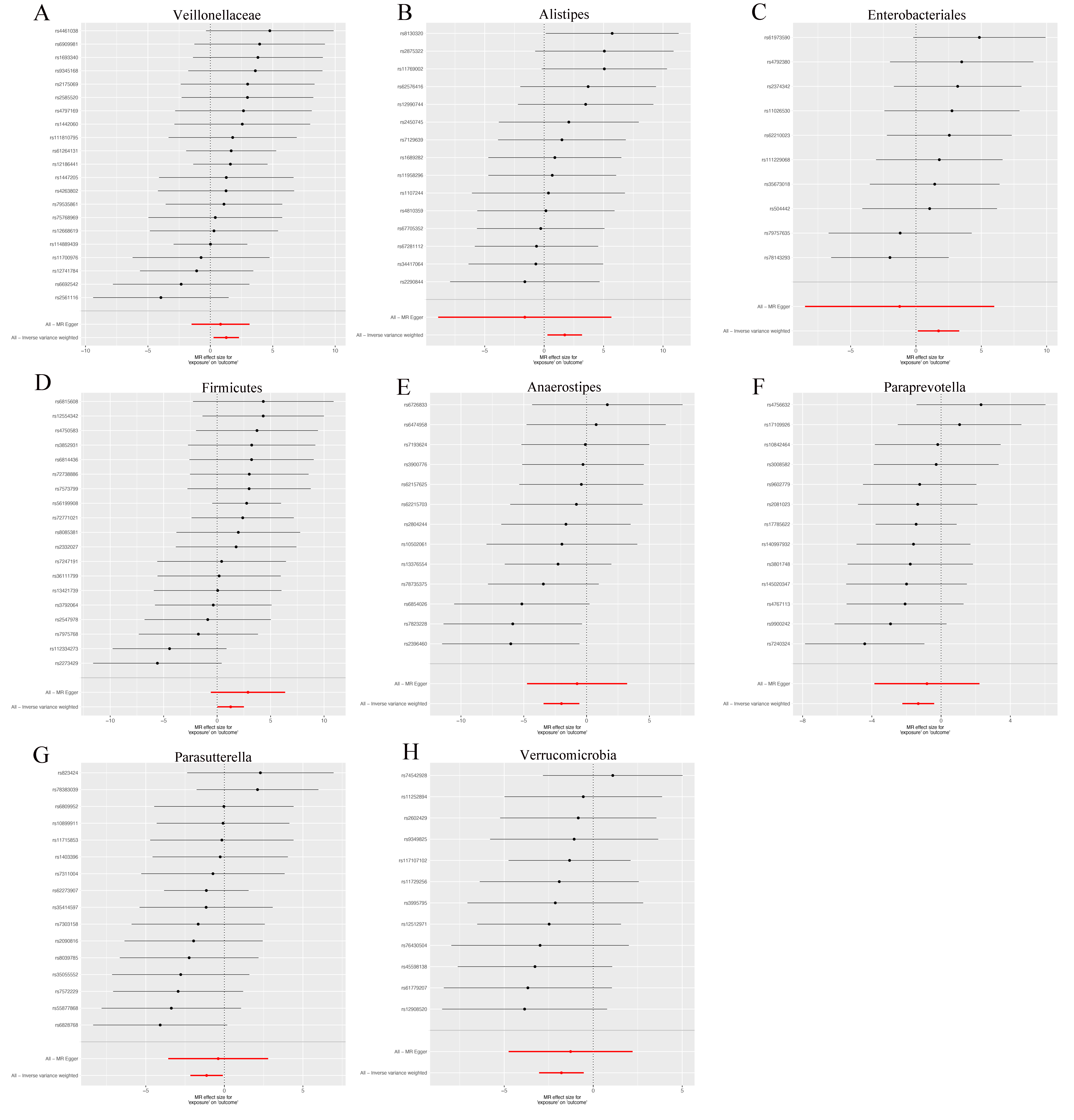

Supplement: Supplementary Figure 1 — Forest plot of the causal effects of gut microbiota associated single nucleotide polymorphisms (SNPs) on intrahepatic cholangiocarcinoma (ICC). (A) Veillonellaceae, (B) Alistipes, (C) Enterobacteriales, (D) Firmicutes, (E) Anaerostipes, (F) Paraprevotella, (G) Parasutterella, (H) Verrucomicrobia. [file Image_1.TIF]

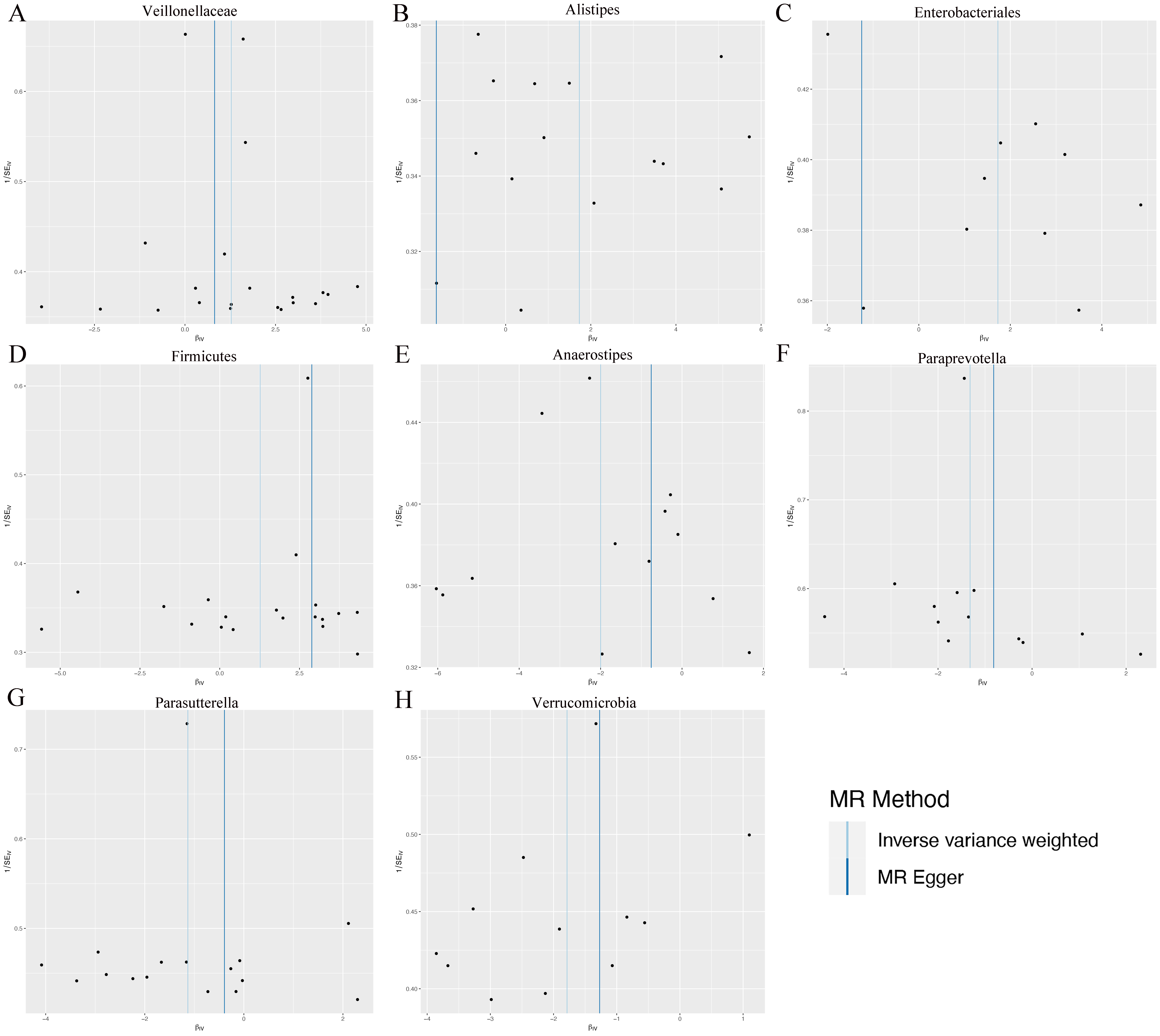

Supplement: Supplementary Figure 2 — Funnel plot showing the relationship between the cause-effect of gut microbiota and intrahepatic cholangiocarcinoma (ICC). (A) Veillonellaceae, (B) Alistipes, (C) Enterobacteriales, (D) Firmicutes, (E) Anaerostipes, (F) Paraprevotella, (G) Parasutterella, (H) Verrucomicrobia. [file Image_2.TIF]
